# On the Nature of the Interactions That Govern COV-2 Mutants Escape from Neutralizing Antibodies

**DOI:** 10.3390/molecules29215206

**Published:** 2024-11-04

**Authors:** Fredy Sussman, Daniel S. Villaverde

**Affiliations:** Department of Organic Chemistry, Faculty of Chemistry, Universidad de Santiago de Compostela, 15784 Santiago de Compostela, Spain; daniel.sussman@rai.usc.es

**Keywords:** COVID-19, COV-2, spike RBD, antibodies, molecular dynamics simulations, epitope

## Abstract

The most fruitful prevention and treatment tools for the COVID-19 pandemic have proven to be vaccines and therapeutic antibodies, which have reduced the spread of the disease to manageable proportions. The search for the most effective antibodies against the widest set of COV-2 variants has required a long time and substantial resources. It would be desirable to have a tool that will enable us to understand the structural basis on which mutants escape at least some of the epitope-bound antibodies, a tool that may substantially reduce the time and resources invested in this effort. In this work, we applied a computational-based tool (employed previously by us to understand COV-2 spike binding to its cognate cell receptor) to the study of the effect of Delta and Omicron mutations on the escape tendencies. Our binding energy predictions agree extremely well with the experimentally observed escape tendencies. They have also allowed us to set forth a structural explanation for the results that could be used for the screening of antibodies. Lastly, our results explain the differences in molecular interactions that govern interaction of the spike variants with the receptor as opposed to those with antibodies.

## 1. Introduction

The outbreak in 2019 of one of the largest pandemics since the Spanish Flu caused untold infections and deaths, as well as economic and social disruption. It was named COVID-19 and the pathogen agent COV-2 by the World Health Organization (WHO) [1]. SARS-CoV-2 continuously undergoes mutations due to changes in the genetic code that usually occur during replication of its genome. The new strains go unchecked because the virus lacks proofreading machinery [2]. In the case of COV-2, the mutants that spread more widely and produced the largest number of deaths were named variants of concern (VOCs) by the WHO. The ones that have proven to have the most lasting effects are the Delta [3] and Omicron [4] VOCs and their variants. The Delta VOC presents two mutations (in its spike receptor-binding domain (RBD)) with respect to the native sequence [3], while the same Omicron spike region includes 15 mutations with respect to the native species [4].

Vaccines and antibodies (Abs) have proved to be the best prevention and therapeutic tools in the fight against this pandemic [5]. Their use has relegated this pandemic to outbreaks of periodic occurrence comparable to those of the Flu.

There has been a vast amount of work devoted to the development of vaccines and Abs [5,6,7]. The spike protein that adorns the spherical particle providing it with its crown (corona) shape has attracted much of the attention as an Ab target since it mediates virus entry into the cell, and hence its infectivity [4]. Two sites in the spike have been targeted by the mABS; the first site is the N terminal Site (NTS) and the other is the RBD (Receptor Binding Domain) [5]. Most of the antibodies targeting the RBD can be classified on the basis of the spike epitopes to which they bind. It has been found that the RBD antibodies bind to four specific RBD epitopes that sometimes display a degree of structural overlap [5,8].

The structures of numerous antibody–spike complexes have been predominantly determined using Cryo-EM [5,7,8,9,10,11,12], providing critical insights into antibody–spike binding. The wealth of structural data has enabled the study of spike–antibody (Ab) complexes using computational methods [13,14,15,16]. Many of these studies have focused on predicting the affinities of a given VOC for a group of antibodies. Another study used an accelerated MD and Ab-initio hybrid MD calculations to identify the most predominant interactions at the atomic level under physiological conditions of the Wuhan (native) RBD with only two (Abs S309 and CR032 Abs, belonging to groups 3 and 4) [13]. Von Bulow et al. performed an extensive MD simulation on the spike trimer, including the glycans that surround it. The structures obtained from the MD trajectories were used to determine which regions of the trimer were sequestered by the sugar shield. They found a correlation between the accessibility to the spike residues and their mutation propensities [14]. Of special interest are attempts at finding algorithms that predict the binding of a given VOC to a set of Abs. Williams and Zhan generated a multi-layer perceptron (MLP) neural network which was used to predict the binding affinity of some of the reported Abs with various VOCs and it may be used in the future to predict binding to emerging Ab’s [15]. Others, such as Chen et al. elaborated a scoring function based on the results of Coarse Grain (CG) dynamics for a set of variants [16].

In this study, we diverge from prior research described above by focusing on the structural characteristics that allow individual mutants to evade antibody binding. This reductionist approach has been previously applied in vitro. A seminal study by Dr. P.D. Wong’s team systematically investigated the effects of each of the 15 mutations in the Omicron variant’s RBD, evaluating their potential to evade antibodies targeting three specific epitopes on the spike protein [7].

Recently, we developed a series of increasingly complex computational protocols to study protein–protein binding affinities, which we initially applied to understanding the Delta and Omicron VOCs and their interaction with the ACE-2 cell receptor. Our analysis revealed that the RBDs of different VOCs engage in distinct types of molecular interactions with the receptor. These findings led us to propose a hypothesis explaining the differing infectivity and lethality of the Delta and Omicron variants [17].

In the present work, we applied our protocol to the study of the binding of a number of RBD spikes mutants to a select group of antibodies that bind to the four types of RBD epitopes. Since the MD calculations will be prohibitive for the study of all single mutants that occur in the various VOCs, we have centered our calculations on the study of a group of selected mutations, which include several of the mutants present in the Omicron variant (K417N, E484A and S371L), as well as the E484K, present in the Alpha and Beta COV-2 variants [18]. We also used our methodology and insights gained from its application to the Delta variant Ab binding with the aim of discerning between opposing binding affinity results (see Results and Discussion, Section 2 for more details).

All of these variants are known to reduce binding to at least some of the antibodies in groups 1–4 [7,18]. The selection of antibodies was based on the availability of antibody–spike complexes in the PDB. A second screening retained only the highest-quality structures, such as those with the highest resolution and no structural gaps. For these reasons, the antibodies selected are CB6 from group 1, Ly-CoV555 from group 2, regn_10987 and S309 from group 3, and CR3022 from group 4 (see Methods, Section 3 for a broader description of the rationale behind the selection of Abs and spike mutations). As a reference, we calculated the binding affinity of the native RBD spike to the set of antibodies listed above.

Several of the calculations were based on single-point methods, such as those using coarse-grained (CG) calculations [16] or single-point evaluations in machine learning protocols [15,19]. However, these approaches overlook the fact that many mutated residues in the spike protein are located in flexible loop regions, which can result in a range of binding score values rather than a single result. Our protocol addresses this by providing the average root mean square deviation of the binding values, allowing us to assess the variability of binding due to the structural flexibility.

Our binding affinity results agree with almost all the in vitro experiments. Dissecting COV-2 variants into single mutations has allowed us to identify structural factors and the nature of the interactions that determine the existence of escape mutations. Comparison of the results obtained in this work for the Ab-spike with those drawn from our receptor-spike studies, has allowed us to clearly show that the binding affinity in both systems is governed by very different molecular interactions.

## 2. Results and Discussion

The scoring affinity values were obtained by the difference:

ΔG = ΔG(Complex) − ΔG(Spike) − ΔG(Ab) (1)

which we applied to a group of structures that resulted from our MD trajectories (see Methods, Section 3 for details).

Table 1 lists binding energy scoring values (as well as their standard deviations (SDs) for the full MD/GBSW calculations. An additional quantity that allowed us to gauge the effect of a mutation is the difference in the resulting binding energy of a mutant (for any given antibody) to the one of the native spike variant.

To validate the results of our protocol, we used the results of the extensive work that was performed in order to assess the susceptibility of multiple-mutation RBD spike variants to various antibodies, as well as evaluating how individual RBD spike mutations impact antibody binding. (see last 2 columns in Table 1) The results have been published not only across individual studies, but there is an extensive compilation that has led to the creation of the Stanford Coronavirus Resistance Database (CoV-RDB; https://covdb.stanford.edu/drms/spike/, accessed 20 October 24), which was designed to house comprehensively curated published data on the neutralizing susceptibility of SARS-CoV-2 variants and spike mutations to monoclonal antibodies (mAbs), convalescent plasma (CP), and vaccine plasma (VP) [20]. 

As can be seen from Table 1, the native to mutant energy differences vary from one group to another, for a given residue mutation. For instance, the E484K mutant affects the LY-CoV555 Ab (that belongs to group 2) but not the CB6 Ab of group 1 (see Table 1). Differences in binding affinities of a mutant spike for different Abs can also be appreciated for several of the mutants that are part of the Omicron VOC, like E484A or K417N. As seen from Table 1, the E484A mutant does not affect the affinity to class 1 Ab Cb6 or class 3 Ab S309 but reduces binding for class 2 Ab LY-CoV555. The variability in the binding score (as given by the SD values) also plays a role. For instance, the Delta VOC presents a strong drop in affinity score when bound to Ab class 2 Ab LY-CoV555 (see Table 1) but not with respect to class 3 Ab regn-1089 (see Table 1). In this latter case, the affinity for the latter variant even displays a rise in binding with respect to the native strain, although that increment is within the standard deviation values of both species. As seen from this example, inclusion of the standard deviation (SD) values enables us to take binding affinity scores as a range of values rather than as a single estimate. Moreover, as seen from Table 1, the SD values seem to depend on the class of antibodies and the mutation studied. For instance, classes 1 and 2 Abs present amongst the largest SD values, while classes 3 and 4 present the lowest.

For the majority of mutants studied here, our predicted binding affinity trends closely match most of the observed data. For mutations associated with the Omicron variant, we compared our results with in vitro findings for each mutant within the Omicron VOC [7], presented as IC50 and IC80 binding titers for antibodies in groups 1–3 [7]. For instance, as seen from Table 1, the results for the binding for spikes RBD that contain K417N and E484A display a strong drop in the predicted binding for Ab BC6 (Class 1) and Ab LY-COv555 (Class 2). These trends are very much in line with experimental results, which observed a very low IC50 and IC80 (>10,000 mg/L) in class for these systems (BC6-K417N and LY-Cov355-E484A) [7]. As predicted by our calculations, the mutations on E484 affect the binding to the CB6 RBD spike much less, with an IC50 of only 26.6 mg/L [7] (See penultimate column in Table 1).

A perusal of Table 1 indicates that classes 3 and 4 are the best-performing Abs as they are less affected by the limited set of mutations studied here. For instance, mutants K417N, E484K, and E484A seem to lower binding to either CB6 or LY-COV555 (of classes 1 and 2, respectively), while they affect much less the binding to REGN-10987 and S309 of class 3, as well as CR3022 of class 4. The superior efficacy (predicted by our calculations) for the latter sets of Abs is in line with known experimental data [5]. In the case of the binding of the COV-2 Delta variant binding to class 2 LY-CoV555 Ab and class 3 regn-10987, our results indicate that this double mutant evades the first Ab (see Table 1), in agreement with experimental results [20]. For the latter Ab, there appears to be a range of conflicting results regarding its binding to the Delta variant. Some show a substantial reduction in affinity [21], while others indicate that the binding to the Delta spike does not reduce binding to a great extent [22]. Our results for Delta variant binding to regn-10987 is in agreement with this latter outcome. Our structural analysis has allowed us to back and explain this outcome (see below).

**Table 1:** Average binding affinities (MD/GB), SD and experimental results.

AB (Class)	Mutation	ΔG (SD)	ΔΔG ^(B)^	Results from Ref. [7] ^(C)^	Experimental Database Ref. [22] ^(D)^
CB6 (class1)	native	−135.0 (14.3) ^(A)^	0.0		-
	K417N	−89.4 (2.3)	−45.6	>10,000	≥25
	E484A	−131.8 (11.3)	−3.2	26.6	<5
	E484K	−133.2 (3.2)	−1.8		<5
Ly-CoV555 (class2)	native	−134.6 (3.5)	0.0		-
	K417N	−110.2 (3.7)	−24.4	3.5	<5
	E484A	−83.2 (5.6)	−51.4	>10,000	≥25
	E484K	−102.4 (17.9)	−32.2		≥25
	Delta	−83.0 (6.5)	−51.6		≥25
regn 10987 (class3)	native	−46.5 (2.1)	0.0		-
	K417N	−45.8 (2.3)	0.7		<5
	Delta VOC	−53.0 (5.8)	−6.5		<5
S309 (class3)	native	−94.9 (3.5)	0.0		-
	K417N	−100.2 (4.0)	−5.3	23.9	<5
	E484A	−91.2 (3.2)	3.3	25.8	<5
	S371L	−93.5 (4.9)	1.4	>10,000	5–25
CR3022 (Class4)	native	−116.5 (4.5)	0.0		-
	K417N	−117.3 (6.5)	0.8		<5
	E484A	118.8 (4.4)	−2.3		<5

^(A)^ Average binding energy and standard deviation in Kcal/mol; ^(B)^ Differences with respect to the native variant; ^(C)^ Titers for IC80 neutralization ng/mL; ^(D)^ Susceptibility binding reduction fold.

### 2.1. Mutation Location and Escape Mutations

**Figure 1:**
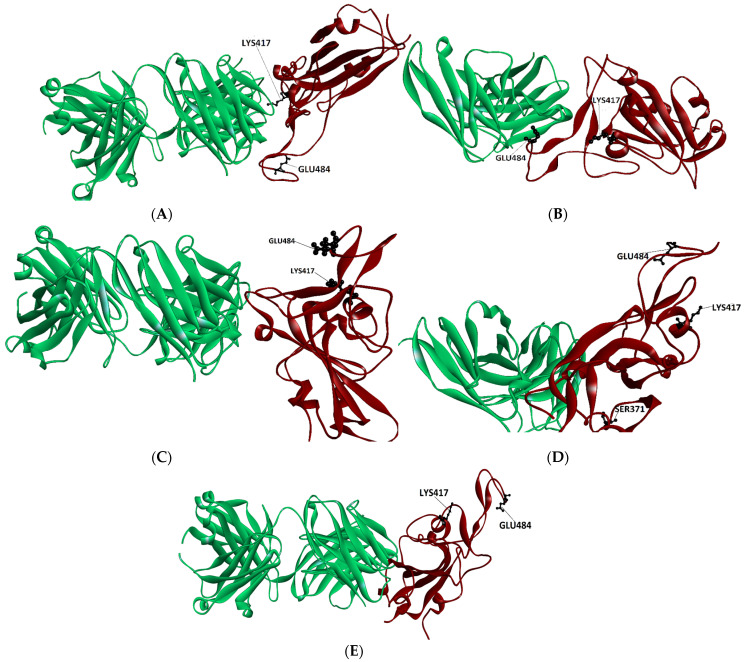
Location of RBD spike sites when bound to class 1 Ab CB6 (**A**), when bound to class 2 Ly-CoV555 (**B**), when bound to class 3 Ab regn-10987 (**C**), when bound to Class 3 Ab S309 (**D**), and to class 4 Ab CR3022 (**E**). Antibodies are depicted in green and spikes in brown.

**Figure 2:**
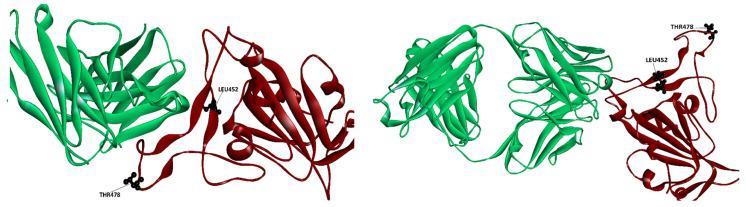
Location of the mutations that comprise the Delta VOC when the RBD spike is bound to class 2 Ab Ly-CoV555 (**left panel**) and when bound to class 3 Ab regn 10987 (**right panel**). Ab is colored in green and spike in brown.

To find the structural features that underpin the escape trends discussed above, we analyzed the data presented above in relation to the structural characteristics of the complexes studied. A feature that the results brought to our attention is that that very different mutants of the same RBD site (see, for instance, E484) produce a strong reduction in affinity towards class 1 Ab, (CB6) and towards class 2 Ab, Ly-CoV555, in spite of the different nature of the mutants (A484 and K484). This result would indicate that the nature of the mutated side chain may not be the most crucial factor in determining the binding affinity trends. A feature that may be a good candidate factor in decreasing the affinity of the mutated spike is the location of mutated residue in the RBD spike with respect to the Ab interface. To evaluate this issue, we have drawn the structures of the complexes studied here, highlighting the position of the residue to be mutated. (see Figure 1 and Figure 2).

At first glance these figures indicate clearly that the spike mutations that are closer to the interface with the antibody are those which lower its affinity for it, and hence may escape antibody binding. For instance, residues K417 as well as E484 are close to the interface with Ab LY-CoV555 (see Figure 1B) and their respective mutations K417N, E484A and E484K, are predicted (by our protocol) to reduce the affinity scores (see Table 1). The same residues (K417 and E484) are the furthest away from the antibody interface in those Ab’s of class 3 (regn 10987, S309) and class 4 (CR3022). (see Figure 1C–E). The respective mutant RBD spike binding affinity to these antibodies is not very altered by these mutations (See Table 1).

As seen from Figure 1A, the E484 residue location is not as far away from the interface with CB6, as it is from the boundary of the class 3 and 4 antibodies (see Figure 1D,E). Hence, a drop in affinity for mutants in the A484 mutants would be expected. Nevertheless, a search for residue neighbors indicates that the nearest antibody atom is not closer than 7 Å, indicating that this residue does not belong to the interface, an outcome that explains our binding results.

One of the results that appears inconsistent with experimental findings is the binding affinity score of the S371L spike mutation. The experimental IC50 and IC80 values indicate a substantial drop in the binding affinity S309 antibody, with titers for IC50 > 10,000 mg/L [7]. Our predicted binding affinity scoring function results indicate that its binding affinity should be in the same range as the native spike. This result is backed by the location of the residue (see Figure 1D), where the residue is seen far away from the binding interface. Another factor that may affect the binding affinity of the S371L spike mutant is the presence of the glycans found in the Ab-spike structure. Nevertheless, examination of the Cryo-EM structure (pdb: 7SOC) indicates that the closest glycan is at 6 Å from the L371 residue, making its influence improbable.

Different experimental setups may be important in this regard, since they may lead to diverging binding affinity results. Search of the CoV-RDB database [20], displays a different result for the S371L mutant bound to S309 Ab. It was found that this mutant only produces a 5–25-fold reduction in affinity, an outcome that is more in line with our predictions.

Studies on the Delta VOC have found that the **only** mutant of the pair that lowers binding is the L452R [21]. The rationale behind this outcome can be found in Figure 2, which displays the location of the single location mutations for the Delta VOC when bound to class 2 Ab Ly-CoV555 and Class 3 Ab regn 10987. As seen from the first picture, one of the mutants (L482R) is found to be in the epitope for Ab Ly-CoV555, which explains the lowering of the binding score affinities seen in Table 1 and Table 2. In the case of class 2 Ab Ly_Co555, both residues are far away from the Ab interface; this supports the possibility that the Delta mutations do not affect binding to this Ab, in support of the results found by Takuya et al. [22], rather than those of Patrick et al. [21].

Table S1 provides a detailed list of spike protein mutants within 3.6 Å and 5 Å of the four antibodies studied. A comparison between this table and the affinity results in Table 1 and Table 2 reveals that only certain mutated residues within 3.6 Å of a given antibody qualify as escape mutations. This suggests that most mutations in variants such as Omicron and Delta do not substantially contribute to antibody escape. This result can be explained by several factors. On one hand, the mutant spike residue must balance two different factors. A very infective variant should not only lower the affinity for some of the Abs, it also should keep or sometimes enhance its affinity for the ACE2 cognate entry receptor, a double task that may not be possible for all spike mutants located at the epitope. For instance, the larger number of mutations found in the Omicron VOC (over the one in the Delta variant) helps maintain its receptor-binding affinity, while lowering affinity to a wide range of antibodies [23]. Other research groups have proposed very different rationales for the rather discrete number of observed escape mutations. Von Bulow et al. postulated that the existing glycans provide a steric ”protection” for some of the epitope residues [14]. Hence, only residues that are not structurally glycan-shielded would tend to enhance its mutation probability.

Most of the structural studies performed on Ab-spike structures were performed using cryo-EM experiments, which produce low-resolution structures for large-size systems. We have found out that optimization protocols are very necessary, sometimes to locate the residues in the Ab–spike interface. This is the case of the spike mutant K417N when bound to Ab class 1 CB6. Originally, the N417 residue is beyond a 5 Å radius from the Ab. Only after undergoing the MD protocol, this residue gets into van der Waals contact with the Ab.

### 2.2. Nature of the Interactions of the Spike Mutants with Their Cell Receptor and Neutralizing Abs

Understanding the molecular interactions governing viral entry into host cells and neutralizing antibody binding is critical for explaining viral infectivity. Early studies focused on the higher affinity of the SARS-CoV-2 spike protein compared to the SARS-CoV-1 spike, which contributes to the increased infectivity of SARS-CoV-2. Bai and Warshel proposed that this higher affinity is due to stronger long-range electrostatic interactions generated by the SARS-CoV-2 spike [24]. In a previous study, we found that the Delta variant of SARS-CoV-2 increases its binding affinity to the receptor by promoting an increase in the contact area between the spike and the receptor, despite the fact that the mutations defining this variant do not directly interact with the receptor. Conversely, our results indicated that the Omicron variant exhibits a distinct binding behavior. Its affinity for the receptor is driven by long-range electrostatic interactions and entropic factors, rather than direct contact [17].

To discern the nature of the Ab–spike interactions, we applied (as part of our battery of methods) the Buried Surface Area (BSA) upon binding protocol, starting from structures obtained from our MD simulations. The results are listed in Table 2. Comparison of these results with those of full MD/GBSA calculations (see Table 1) indicates that all the trends observed with the more extensive MD/GBSW (see Table 1 ), are reproduced by this much simpler approach, a result that indicates that the Ab–spike van der Waals contacts between some of the residues located in the spike epitope with those of the Ab paratope are responsible for the binding trends observed in the various classes of Abs.

One key factor that may explain the difference in the interactions between the spike-Omicron–receptor complex, compared to the Ab–spike system, is the net charge difference between the ACE-2 receptor and the antibodies. The ACE-2 receptor is highly acidic, carrying a strong negative charge (−23), while the antibodies have a small net positive charge (+3 to +5). The significant negative charge of the receptor likely enhances long-range electrostatic interactions with residues outside the direct protein–protein interface, as observed with the Omicron spike.

## 3. Methods

All the spike–antibody structures used in this work were downloaded from the Protein Data Bank (PDB) [25]. The pdb structures and their list of antibodies are itemized in Table 3.

Although numerous Ab–spike structures have been described, primarily through cryo-EM, many Abs still lack pdb structures of their complexes with any spike protein. Moreover, some structures have structural gaps. For this reason, we selected a finite set of available structures that do not present any sequence voids. Mutations were selected based on their ability to significantly reduce the affinity for at least one antibody within the given class, while retaining some binding to others. We hoped that this choice would enable us to identify the features that produce escape mutants.

As mentioned before, we used a reductionist approach to understand the main features behind escape mutations. For this sake we selected (for this study) single mutants that belong to the Omicron variant that present a reduced affinity for at least one of the Abs, while keeping it for others. We also selected the E484K mutation (that belongs to the Alpha and Beta VOCs) specifically to determine the effect of the side chain character on binding on the E484 site. Finally, we included the Delta double mutant as an application of our results to distinguish between competing abiding results.

Some of the PDB structures listed above contained mutated spikes. To produce a native spike, we replaced the mutated residues for those of the native variant spike, using Discovery Studio [26]. This was the case of the 7ZJL structure, which contains a Delta spike and 7SOC which contains a Kappa spike. The various spike mutants were generated (starting from the native containing spikes) as well by Discovery Studio [26]. When a cryo–EM structure had more than one Ab, we kept the one we were interested in. The input files for the molecular dynamics simulations were generated by the CHARMM-GUI Web interface [27]. The force field used in all simulations was CHARMM_36 [28]. To avoid the use of all atom solvent, we used the Generalized Born with the switching (GBSW) implicit solvation function [29]. In order to mimic the friction provided by the solvent, we used Langevin dynamics [30] with a friction factor (fbeta) of 20. In all cases, the time step was 2 femto-seconds and a cutoff of 18 Ǻ for non-bonded interactions was used.

The dynamics protocol was divided into two stages: In the first one, a dynamic trajectory of 2.5 ns was performed with an initial heating, equilibration, and preliminary production steps that added up 0.5 ns, followed by 10 steps, each one of 0.2 ns. The structures saved after each of these steps was cooled down to 50K in two stages. The final frame of each trajectory (a total of 10) was saved for analysis of the spike–receptor interaction energies, which was evaluated using the same MM/GBSW protocol as the dynamics runs.

The simplest alternative to an MD/GB protocol is the evaluation of the Buried Surface Area (BSA) generated by the protein–protein association. This quantity has been shown to be a primary descriptor of the binding affinity or interaction energy of two macromolecular entities. A first-order approximation assumes that the BSAs could be proportional to the binding energies with a proportionality constant of 0.025 Kcal/mol per Å^2^ of surface protein removed from contact with water [31].

### Methods Associated Content

The input files for the MM/MD simulations (which included the parameter, connectivity files, coordinate files, etc.) were obtained through the input generator option CHARMM-GUI interface https://www.charmm-gui.org/.

The MM/MD calculations were carried out by CHARMM version 46. The software can be found at https://www.charmm.org/archive/charmm/showcase/news/free-charmm/ (accessed on 30 October 24) and the documentation at https://www.charmm.org/archive/charmm/documentation/ (accessed on 30 October 24)

The parameter files and connectivity library can be found at https://github.com/fsussman/TOPPAR1 (accessed on 30 October 24)

The binding free energy calculations based on the BSA approach for the frames resulting from the MD trajectories was performed with the free version of Discovery Studio V3.5, which could be found at: https://discover.3ds.com/discovery-studio-visualizer-download?gclid=CjwKCAjwu4WoBhBkEiwAojNdXpgnprVjkxLBhnSIdL1mRc6-7_twnRb26LNEP0y1-2G68t65nrpqSRoCH2wQAvD_BwE (accessed on 30 October 24). Documentation is available for every task in the graphic interface.

## 4. Conclusions

In this work, we used a reductionist approach to the understanding of the structural features that contribute the most to promoting escape mutations. For this sake, we studied single mutations that are part of the Omicron VOC as well as a E484K single mutant of the Alpha and Beta variants as well as the double mutation found in the Delta COV. A multi-protocol approach developed by us to study the binding of COV-2 Delta and Omicron RBD spike variants to the ACE-2 receptor [17] was applied to the nature of spike–Ab interactions for Abs belonging to the four known classes. Our results coincide with all experimental results. In some cases (S371L mutant or Delta VOC) where different studies yield contradictory results, our calculations (supported by our structural hypothesis) enabled us to choose one of the experimental outcomes as the most probable. The reduction in affinity displayed by some spike mutants seems to depend mostly on their location, with those belonging (or being close) to the epitope contributing the most to lower affinity for the various Abs. A simple BSA approach reproduces the rankings of the full MD/GBSW approach indicating that escape mutations arise from the disruption of interactions, including van der Waals forces, between residues in close contact. This insight was used to discern between contrary results found for the Delta binding affinity for Ab Regn 10987.

Our work opens avenues of research for the exploration of novel Abs. We have shown that our protocol shows promise in predicting escape from Abs. It also is able to discern between competing experimental results, and it could be useful in the search for novel Abs. For instance, novel artificial intelligence protocols such as AlphaFold 2 could predict the complex between a given COV-2 spike and a candidate Ab [32]. Then, Buried Surface Area calculations can be performed (after structural optimization) to predict the binding affinity of a given Ab–spike complex, which results from the Ab–spike complex structure prediction.

## Figures and Tables

**Table 2:** Buried Surface Accessibility (BSA) based score binding energies.

AB (Class)	Mutation	ΔBSA (sd) ^(A)^	ΔΔBSA ^(B)^
CB6 (class1)	native	−58.5 (3.4)	0.0
	K417N	−35.4 (0.4)	−23.1
	E484A	−61.1 (0.7)	2.6
	E484K	−56.6 (0.7)	1.9
Ly-CoV555 (class2)	native	−55.0 (1.4)	0.0
	K417N	−49.6 (0.7)	−5.4
	E484A	−41.9 (0.4)	−13.1
	E484K	−43.7 (3.3)	−11.3
	delta	−37.4 (0.8)	−17.6
regn 10897 (class3)	native	−30.9 (0.6)	0.0
	K417N	−31.3 (0.3)	0.4
	delta VOC	−29.3 (1.3)	−1.6
S309 (class3)	native	−39.1 (0.6)	0.0
	K417N	−41.2 (0.7)	2.1
	E484A	−37.8 (1.2)	1.3
	S371L	−45.8 (2.4)	6.7
CR3022 (class4)	native	−50.1 (1.0)	0.0
	mutant K417N	−48.4 (1.2)	−1.7
	mutant E484A	−49.7 (1.6)	−0.4

^(A)^ Calculated Ab–spike score binding energy value based on BSA calculations; ^(B)^ Difference in scoring binding energy from the native strain.

**Table 3:** PDB entries and antibodies used in this work.

PDB Entry	Ab Name
7C01	CB6
7L3N	LY_Cov555
7ZJL	REGN_10987
7SOC	S309
6ZLR	CR3022

## Data Availability

Data are contained within the article and Supplementary Materials.

## Supplementary Materials

The following supporting information can be downloaded at: https://www.mdpi.com/article/10.3390/molecules29215206/s1, Table S1: Spike contacts with antibody at 3.6 Å and 5 Å.
