# Supplementary material for: On the Nature of the Interactions That Govern COV-2 Mutants Escape from Neutralizing Antibodies
Source: Molecules. 2024 Nov 4;29(21):5206. doi: 10.3390/molecules29215206 (PMC11547327; doi:10.3390/molecules29215206)
Supplement: Supplementary file 1 [file molecules-29-05206-s001.zip › molecules-3247178-supplementary.pdf]

SUPPORTING INFORMATION

On the Nature of the Interactions That Govern COV-2 Mutants Escape from Neutralizing Antibodies

Fredy Sussman & Daniel S. Villaverde

Department of Organic Chemistry,
Faculty of Chemistry, Universidad de
Santiago de Compostela, 15784
Santiago de Compostela, Spain

e-mail to fredy.sussman@usc.es (F.S)

TABLE S1 Spike contacts with antibody at 3.6 Å and 5 Å identifying those in VOC's

ANTIBODY	3.6 Å	5.0 Å
CB6	ARG403, ARG408, GLN409, ASP420, TYR421, LEU455, LYS458, ASN460, ALA475, GLY476, PHE486, ASN487, TYR489, **GLN493** (mut omicron), **TYR505** (mut omicron)	**SER477** (mut omicron), PHE486, ASN487, **GLN493** (mut omicron), SER494, TYR495, THR500, **ASN501** (mut omicron), GLY502, VAL503, GLY504, GLY476, ALA475, GLN474, TYR473, ASN460, SER459, LYS458, ARG457, PHE456, LEU455, TYR453, TYR421, ASP420, **LYS417** (mut omicron), GLY416, THR415, GLN409, ARG408, GLU406, ASP405, ARG403, **TYR505** (mut omicron)
ly_Cov555	TYR449, **LEU452** (mut delta), TYR453, ASN481, GLY482, VAL483, **GLU484** (mut omicron) GLY485, PHE486, PHE490, LEU492, **GLN493** (mut omicron) SER494	PHE486, GLY485, **GLU484** (mut omicron) VAL483, ASN481, **THR478** (mut delta), TYR449, ASN450, **LEU452** (mut delta) TYR453, TYR351, PHE456, THR470, **GLN493** (mut omicron) SER494, PHE490, LEU492, GLY482
REGN_10987	ASN450, TYR449, ASN448, GLY447, VAL445, LYS444, LEU441, ASN440, ASN439, **GLN498** (mut omicron), PRO499, THR500, GLN506	THR345, ASN439, ASN440, LEU441, SER443, LYS444, VAL445, **GLY446** (mut omicron) GLY447, ASN448, TYR449, ASN450, **GLN498** (mut omicron), PRO499, THR500, **ASN501** (mut omicron), GLY502, GLN506
CR3022	ASN334, LEU335, GLY339, GLU340, ASN343, THR345, ARG346, LYS356	THR333, ASN334, LEU335, **CYS336** (mut omicron), PRO337, GLY339, GLU340, VAL341, ASN343, ALA344, THR345, ARG346, ASN354, LYS356, ARG357, ILE358, SER359,

		ASN360, CYS361, LEU441, ARG509
6zlr	SER371, PHE374, PHE377, LYS378, CYS379, TYR380, GLY381, SER383, PRO384, LYS386, THR430, LEU517	LEU517, HIS519, ASP428, PHE392, PHE429, GLY381, TYR380, CYS379, LYS378, PHE377, THR376, **SER375** (mut omicron), PHE374, ALA372, ASN370, LYS386, TYR369, THR385, **SER371** (mut omicron), **SER373** (mut omicron), PRO384, PHE515
